# 2-Methoxyestradiol Induces Mitotic Arrest, Apoptosis, and Synergistic Cytotoxicity with Arsenic Trioxide in Human Urothelial Carcinoma Cells

**DOI:** 10.1371/journal.pone.0068703

**Published:** 2013-08-13

**Authors:** Kuan-Lin Kuo, Wei-Chou Lin, I-Lin Ho, Hong-Chiang Chang, Ping-Yi Lee, Yuan-Ting Chung, Ju-Ton Hsieh, Yeong-Shiau Pu, Chung-Sheng Shi, Kuo-How Huang

**Affiliations:** 1 Department of Urology, National Taiwan University Hospital, College of Medicine, National Taiwan University, Taipei, Taiwan; 2 Department of Pathology, National Taiwan University Hospital, College of Medicine, National Taiwan University, Taipei, Taiwan; 3 Graduate Institute of Clinical Medical Sciences, Chang-Gung University, Taoyuan, Taiwan; 4 Department of Medical Research, Chang-Gung Memorial Hospital, Chiayi, Taiwan; Wayne State University, United States of America

## Abstract

2-Methoxyestradiol (2-ME), an endogenous derivative of 17β-estradiol, has been reported to elicit antiproliferative responses in various tumors. In this study, we investigated the effects of 2-ME on cell viability, proliferation, cell cycle, and apoptosis in human urothelial carcinoma (UC) cell lines. We used two high-grade human bladder UC cell lines (NTUB1 and T24). After treatment with 2-ME, the cell viability and apoptosis were measured by MTT assay and flow cytometry (fluorescence-activated cell sorting), with annexin V-FITC staining and propidium iodide (PI) labeling. DNA fragmentation was analyzed by agarose gel electrophoresis. Flow cytometry with PI labeling was used for the cell cycle analyses. The protein levels of caspase activations, poly (ADP-ribose) polymerase (PARP) cleavage, phospho-histone H2A.X, phospho-Bad, and cell cycle regulatory molecules were measured by Western blot. The effects of the drug combinations were analyzed using the computer software, CalcuSyn. We demonstrated that 2-ME effectively induces dose-dependent cytotoxicity and apoptosis in human UC cells after 24 h exposure. DNA fragmentation, PARP cleavage, and caspase-3, 7, 8, 9 activations can be observed with 2-ME-induced apoptosis. The decreased phospho-Bad (Ser136 and Ser155) and mitotic arrest of the cell cycle in the process of apoptosis after 2-ME treatment was remarkable. In response to mitotic arrest, the mitotic forms of cdc25C, phospho-cdc2, cyclin B1, and phospho-histone H3 (Ser10) were activated. In combination with arsenic trioxide (As_2_O_3_), 2-ME elicited synergistic cytotoxicity (combination index <1) in UC cells. We concluded that 2-ME significantly induces apoptosis through decreased phospho-Bad and arrests bladder UC cells at the mitotic phase. The synergistic antitumor effect with As_2_O_3_ provides a novel implication in clinical treatment of UC.

## Introduction

Bladder urothelial carcinoma (UC) ranks fourth in men and eighth in women in incidences of cancers in the United States [1]. Metastatic bladder UC has always been a devastating disease. Most patients still die of metastatic disease and the overall median survival is about 1 year. Cisplatin-based chemotherapy is the standard treatment for patients with metastatic bladder UC [2]. However, approximately 30–50% of patients develop chemoresistance which will eventually lead to death. Moreover, the chemotherapy-related side effects or toxicities are substantial [3]. Therefore, it is imperative to develop new drugs and novel combination regimens to prolong survival and minimize chemotherapy-related morbidity [4].

2-Methoxyestradiol (2-ME), an endogenous metabolite of 17β-estradiol (E_2_), is present in human urine and blood [5], [6]. Estrogens occurring naturally in the body are metabolized to catecholestrogens (2- and 4-hydroxyestradiol) by cytochrome P450 enzymes. 2-Hydroxy catecholestrogens are further metabolized by catechol-O-methyltransferase to 2-methoxyestradiol [6]. 2-ME was reported to be a promising antitumor drug due to its minimal toxicity and potent inhibition of tumor growth [5], [7].

2-ME has been reported to elicit antitumor effects in various cancers *in vitro* and *in vivo* [5], [8], [9], [10], [11], and has also been used in a number of preclinical and clinical studies for the treatment of solid tumors [5], [12]. However, few studies have been conducted to investigate the cytotoxic effect of 2-ME on human bladder UC cells. A previous study indicated that 2-ME induced G2/M cell cycle arrest and subsequent apoptosis [11], [13], [14], [15]. However, the exact mechanism of 2-ME-induced cytotoxicity in tumor cells remains unclear [5], [7]. In this study, our aim was to examine the efficacy and underlying mechanism of 2-ME-induced cytotoxicity in human UC cells. The effect of 2-ME on cell cycle was investigated by analyzing the cell cycle regulatory proteins. We also examined the cytotoxic effect of 2-ME in combination with As_2_O_3_ on UC cells.

## Materials and Methods

### Cell culture

We used three cell lines to perform the experiments: SV-HUC, NTUB1, and T24. SV-HUC, abbreviated from simian virus 40-immortalized human urothelial cells, is an SV40-transformed, immortalized, and non-tumorigenic human urothelial cell line [16]. The T24 cells were derived from a high-grade human urinary bladder carcinoma [17]. The NTUB1 cells were derived from the surgical specimen of a 70-year-old female patient with high grade UC, and were proven to be tumorigenic in nude mice [18], [19], [20]. The SV-HUC and T24 cell lines were obtained from the Bioresource Collection and Research Center (BCRC, Hsinchu, Taiwan). The NTUB1 cell line was kindly provided by Dr. Yeong-Shiau Pu (Department of Urology, National Taiwan University Hospital, Taipei, Taiwan).

The cells were maintained at 37°C in a humidified incubator under 5% CO_2_ atmosphere in RPMI-1640 medium (for NTUB1 cells), Dulbecco's modified Eagle medium (DMEM, for T24 cells), or Ham's F12 medium (for SV-HUC cells), supplemented with 10% fetal bovine serum (HyClone, Logan, Utah, USA), 100 U/mL penicillin, and 100 µg/mL streptomycin. The cell culture media and antibiotics were purchased from Lonza (Walkersville, MD, USA).

### Reagents and antibodies

Arsenic trioxide (As_2_O_3_) and 2-ME were purchased from Sigma Aldrich (St. Louis, MO, USA). Various concentrations of As_2_O_3_ and 2-ME were diluted in the culture media promptly before exposure to the cells. Antibodies for Western blot such as phospho-histone H2A.X (Ser139), Bcl-2 associated death promoter (Bad), phospho-Bad (Ser-136 and 155), poly (ADP-ribose) polymerase (PARP), cleaved PARP, caspase-8 and 9, cleaved caspase-3 and 7, p27, cyclin A2, B1, D1, D3 and E2, cyclin-dependent kinase (CDK) 4, phosphorylated cell division cycle protein 2 (phospho-cdc2) at Tyr15, cdc2, cdc25C, and phospho-cdc25C (Thr48 and Ser216) were purchased from Cell Signaling Technology (Danvers, MA, USA). Anti-CDK2 antibody was obtained from Santa Cruz Biotechnology (Santa Cruz, CA, USA), and anti-phospho-histone H3 (Ser10) antibody was obtained from Merck Millipore (Billerica, MA, USA). Other antibodies against histone H3, GAPDH, and β-actin were purchased from GeneTex (Irvine, CA, USA). All other reagents and chemicals were purchased from Sigma-Aldrich and Serva (Heidelberg, Germany).

### Measurements of cell viability and apoptosis

Cell viability was determined by 3-(4,5-dimethylthiazol-2-yl)- 2,5-diphenyl tetrazolium (MTT, Sigma-Aldrich) assay, using the same methods as the previous study [19]. For the apoptosis assay, the cells were assayed as the previous report and analyzed with Becton Dickinson LSR II flow cytometry (BD Bioscience, San Jose, CA, USA) [19].

### Cell proliferation assay

To determine the effect of 2-ME on cell proliferation, we performed a 5-bromo-2′-deoxyuridine (BrdU) incorporation assay with a commercial quantification kit (Merck Calbiochem, Darmstadt, Germany) and manufacturer's protocol. Briefly, the cells were seeded in 96-well microplates (6000 cells/well) for 24 h and then exposed to 2-ME and DMSO (as non-treated control) for 24 h. During the final 16 h of treatments, BrdU label solution is added to wells of the plate. After fixation with fixative/denaturing solution (provided from kit), the cells were incubated with anti-BrdU monoclonal antibody in antibody diluent (provided from kit) for 1 h at room temperature to bind to incorporated BrdU. Then the cells were washed with Tris-buffered saline with 0.1% Tween-20 (TBST) buffer and incubated with peroxidase goat anti-mouse IgG in the conjugate diluent (provided from kit) for 30 min at room temperature. After washing with TBST, the cells were applied with chromogenic substrate tetra-methylbenzidine (TMB) for 15 min and then added with stop solution (0.16 M sulfuric acid). The absorbance in each well was detected with Biotek μQuant ELISA reader (Winooski, VT, USA) at dual wavelengths of 450–540 nm.

### Cell cycle analysis

After treatments with 2-ME or DMSO (as non-treated control) for 12 h and 24 h, the cells were detached by trypsin-EDTA solution, washed with phosphate buffered saline (PBS), and fixed with 75% methanol at −20°C for 12 h. The samples were centrifuged to remove methanol and then washed with PBS. After removing PBS by centrifugation, the samples were mixed well with FxCycle™ propidium iodide (PI)/RNase staining solution (Invitrogen, Carlsbad, CA, USA) for 30 min at room temperature in dark. Then the DNA content profiles were analyzed with Becton Dickinson LSR II flow cytometry (BD Bioscience).

### Western blot analysis

After various treatments, the cells were washed once with cold PBS and harvested with cell lysis buffer (Cell Signaling Technology) accompanied with scraping and sonication. Then the cell lysates were centrifuged at 14000 rpm for 15 min at 4°C. The supernatants were collected and the concentrations of the proteins were measured via BCA protein assay (Thermo Scientific Pierce, Rockford, IL) according to the manufacturer's instruction. The extracts were mixed with an equal volume of 2× Laemmli sample buffer (Serva) and boiled at 100°C for 5–10 min. The equal quantity of each samples (25–50 µg) were resolved by SDS-PAGE and then transferred to PVDF membranes (GE Healthcare, Piscataway, NJ, USA). The membranes were blocked with 5% BSA/TBST for 1 h at room temperature, and then incubated with primary antibodies in 3% BSA/TBST at 4°C overnight. After incubations with primary antibodies, the membranes were washed three times with TBST for 10 min and then incubated with HRP-conjugated secondary antibodies (Cell Signaling Technology) in 3% BSA/TBST for 1 h at room temperature. After washing three times with TBST for 10 min each, the chemiluminescent signals on membranes were then detected by applying the ECL solution (Millipore, Billerica, MA, USA) and visualized by ImageQuant LAS 4000 system (GE Healthcare). In addition, the relative expression level of target proteins was quantified with Image J (NIH, USA) followed by normalizing to each internal control.

### DNA fragmentation assay

The DNA fragmentation assay was performed according to the manufacturer's instruction of the Quick DNA Ladder Detection Kit (Invitrogen). In brief, after treatments with 2-ME or DMSO (as non-treated control) for 24 h, the cells were detached by trypsin/EDTA solution and washed with PBS. The cell pellets were collected by centrifugation for 5 min at 500× g. After discarding the supernatants, the cell pellets were lysed with TE Lysis Buffer (provided from the kit). Enzymes (provided from the kit), required for eliminating components except DNA, were added to the lysates and incubated for indicated times and temperatures. Ammonium acetate solution and absolute ethanol were then added to allow DNA to precipitate in −20°C for 15 min. The precipitated DNA were harvested by centrifugation at 14000 rpm for 10 min and washed with 70% ethanol followed by centrifuging to get precipitated DNA again. After discarding the supernatants, the air-dried DNA was re-suspended in nuclease-free water. The equal quantity of each samples were loaded onto 1.2% agarose gel electrophoresis and visualized with ethidium bromide staining and UV illumination.

### Combinative drug effects

The combinative effects were determined by the computer software CalcuSyn (version 1.1.1, 1996, Biosoft, Cambridge, UK). The combinative effects of As_2_O_3_ and 2-ME at the combination ratio (1∶10) were subjected to median-effect analysis with the mutually nonexclusive model, as previously described [21], [22]. To generate the combinative effects, we first determined the effects of As_2_O_3_ and 2-ME alone and then in combinations. By combining two agents at graded concentrations, numerous combinative effects of growth inhibition were obtained and analyzed using the CalcuSyn software. For each combinative dose effect (or fraction affected), a combination index was generated. The effects of the combinations were then transformed into and displayed as fraction affected-combination index plots. The combination indexes less than 1, equal to 1, and greater than 1 indicated synergism, additivity, and antagonism, respectively.

### Statistical analysis

The GraphPad Prism® 4 software was used to perform all data analysis. All data were expressed as mean ± SD and analyzed using one-way ANOVA followed by the Bonferroni post hoc test, with values of p<0.05 considered statically significant.

## Results

### 2-ME inhibits cell viability and induces apoptosis in human UC cells

**Figure 1:**
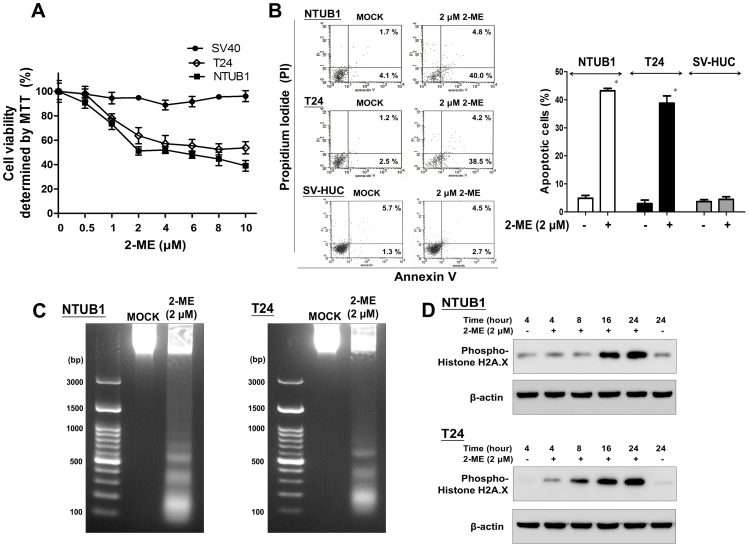
2-ME reduces cell viability and induces apoptosis as well as DNA fragmentation in UC cells, but not in SV-HUC cells. (A) Cells were treated with various concentrations of 2-ME for 24 h. Cell viability was assessed by MTT assay. (B) Cells were exposed to mock (DMSO) and 2-ME (2 µM) for 24 h. Apoptotic cells were analyzed by FACS flow cytometry with propidium iodide (PI) and annexin V-FITC staining. The lower-right panel presented annexin V-positive cells (early apoptotic cells); the upper-right panel presented late apoptotic cells with membranes permeable to PI and annexin V staining. Quantitative analyses of total apoptosis (early and late) population following 2-ME (2 µM) and mock (DMSO) treatments were presented. In (A) and (C), data are presented as means ± SD of three independents experiments. * p<0.05 is interpreted to be significant as compared with control. (C). Agarose gel electrophoresis of DNA extracted from UC cells treated with 2-ME (2 µM) for 24 h. Left lane: DNA marker; Middle lane: treatment with mock (DMSO); Right lane: treatment with 2-ME (2 µM) (D). The total cell lysates were harvested and analyzed by Western blot with specific antibodies against phospho-H2AX.

We first assessed the effects of 2-ME on the viability of human UC cell lines (NTUB1 and T24) and SV-HUC cells by using the MTT assay. As shown in Figure 1A, 2-ME reduced cell viability in a dose-dependent manner in the NTUB1 and T24 cells after 24 h treatments. However, the inhibitory effect on SV-HUC cells was not significant. We then assessed the apoptotic effects of 2-ME on two UC and SV-HUC cells by using annexin V-FITC/PI labeling flow cytometry. As shown in Figure 1B, 2-ME induced apoptosis in the UC cells, but not in the SV-HUC cells after 24 h treatment.

### 2-ME induces DNA fragmentation and phosphorylation of histone H2A.X in human UC cells

We then examined the agarose gel electrophoresis of DNA extracted from T24 and NTUB1 cells treated with 2-ME (2 µM) for 24 h. The results showed a typical “DNA ladder” of apoptosis (Figure 1C). Additionally, as we know, phosphorylation of histone H2A.X is one of the first cellular responses when double-strand breaks are induced [23]. The total cell lysates analyzed by Western blot also showed that 2-ME can induce phosphorylation of histone H2A.X in a time-dependent manner (Figure 1D). Moreover, the activation of histone H2A.X in SV-HUC cells after 2-ME treatment was not significant (Figure S1A).

### 2-ME induces caspases activation and PARP cleavage in human UC cells

**Figure 2:**
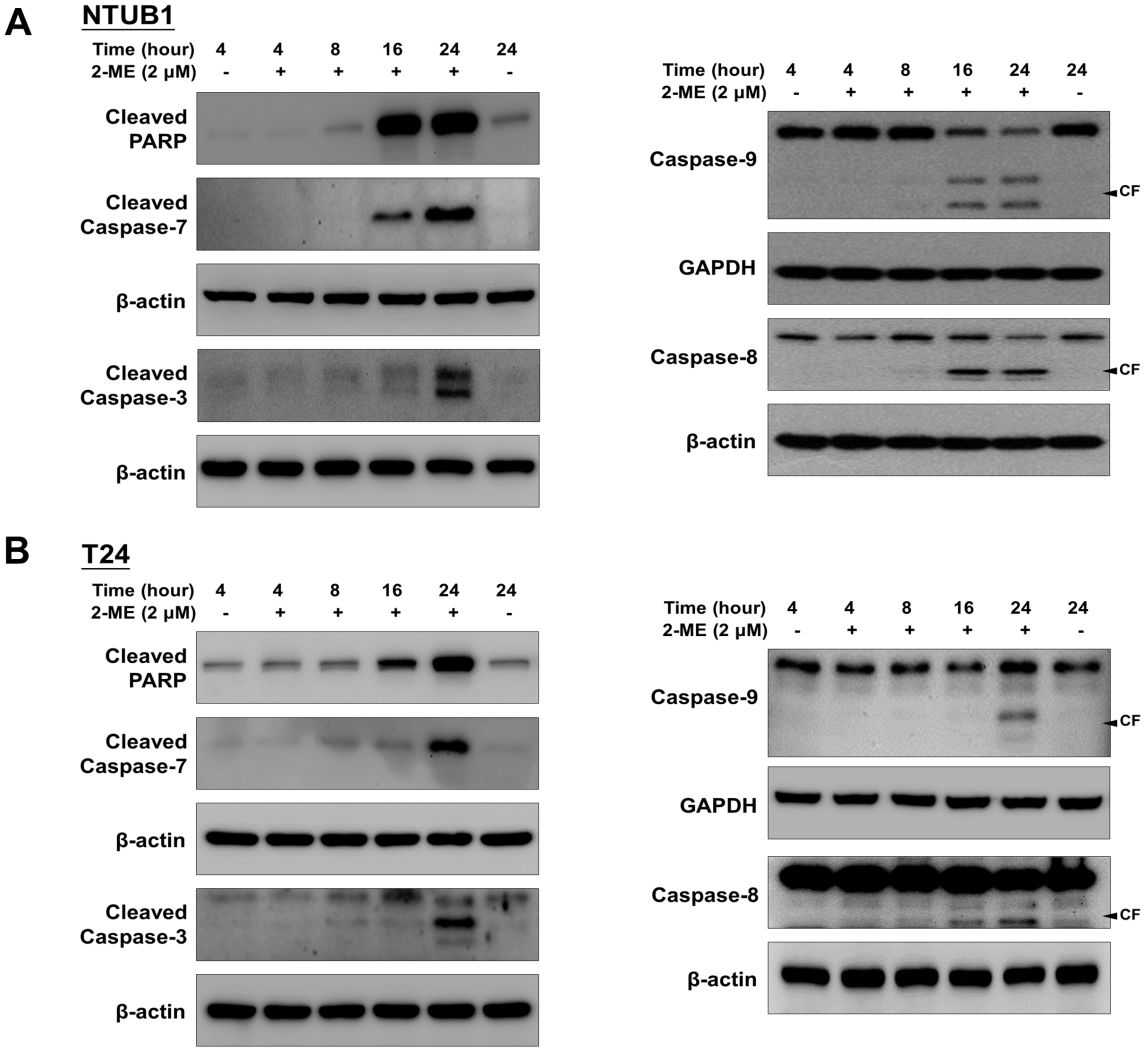
2-ME induces caspase activations and PARP cleavage in UC cells. (A) NTUB1 and (B) T24 cells were treated by 2-ME (2 µM) at different time point. The total cell lysates were harvested and analyzed by Western blot with specific antibodies against caspase-8, 9, cleaved caspase-3, 7 and PARP. CF is the abbreviation of cleaved form. Results shown are representative of at least three independent experiments.

We then assessed the activations of the caspases and PARP cleavage in the total cell lysates by Western blot after 2-ME treatments. As shown in Figure 2, the apoptotic effects of 2-ME on T24 and NTUB1 cells were associated with the activations of caspases (3, 7, 8, and 9) and PARP cleavage (Figure 2A and 2B). 2-ME could not induce activations of caspases and PARP cleavage in SV-HUC cells (Figure S1B).

### 2-ME downregulates phosphorylated Bad at Ser136 and 155

**Figure 3:**
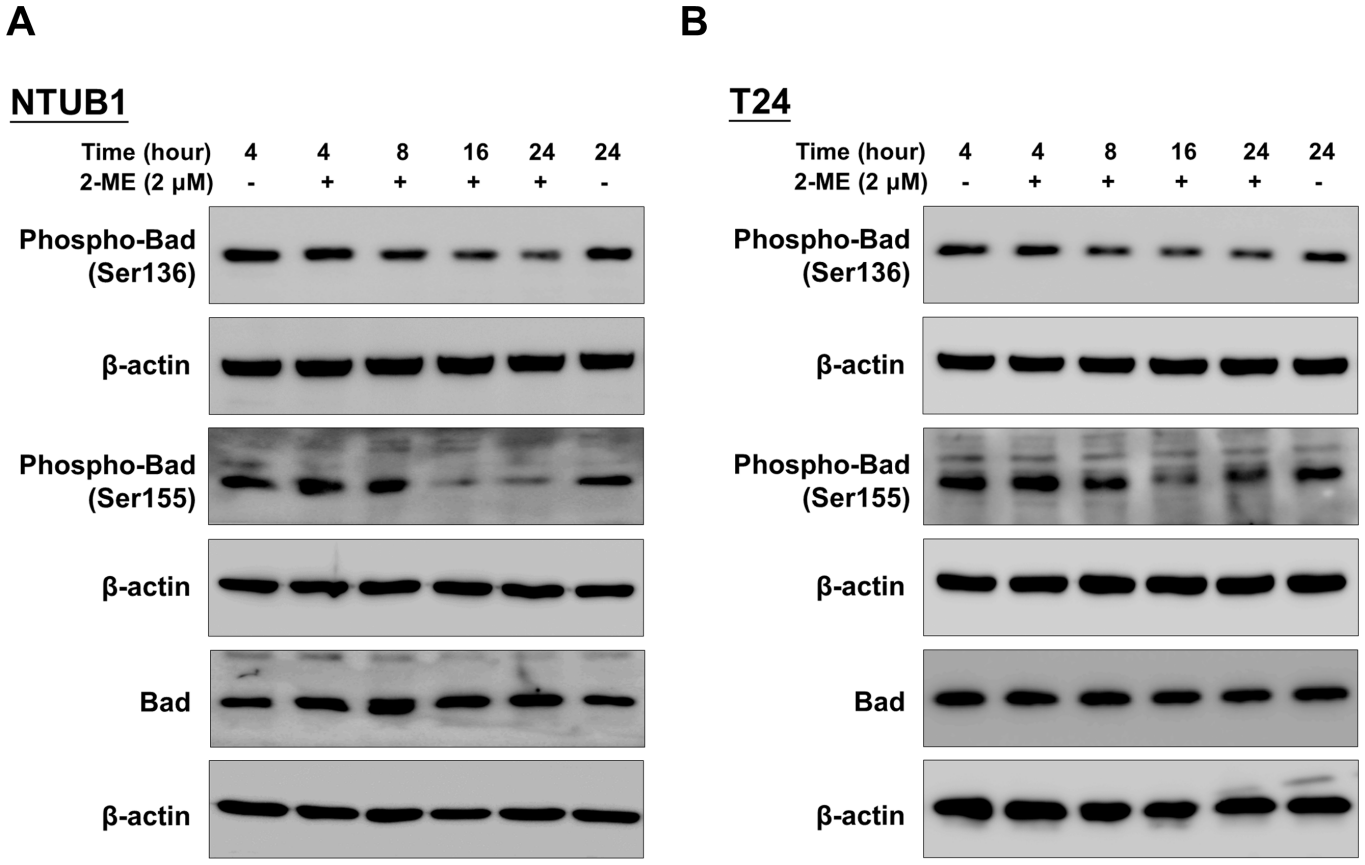
2-ME induces downregulation of phospho-BAD at Ser136 and Ser155. (A) NTUB1 (B) T24 cells were exposed to 2-ME (2 µM) for 4 to 24 h. Cell lysates were analyzed by Western blot with anti-phospho-BAD antibodies at Ser136 and 155.

Previous studies have indicated that non-phosphorylated B-cell lymphoma 2 (Bcl-2) and Bad protein induce apoptosis through heterodimerization with B-cell lymphoma-extra large (Bcl-xL) protein or Bcl-2, thereby allowing the proapoptotic proteins, Bcl-2 homologous antagonist killer (Bak) and Bcl-2–associated X (Bax), to induce the release of cytochrome c and then caspase activations [18]. The phosphorylated Bad lost the proapoptotic effect via being sequestered in the cytosol by binding to 14-3-3 [24]. We then next examined the effects of 2-ME on the expressions of phosphorylated Bad at Ser136 and Ser155. As shown in Figure 3, 2-ME decreased phospho-Bad at Ser136 and Ser155 in a time-dependent manner, thereby inducing apoptosis.

### 2-ME inhibits cell proliferation and induces cell cycle arrest at the G2/M phase in UC cells

**Figure 4:**
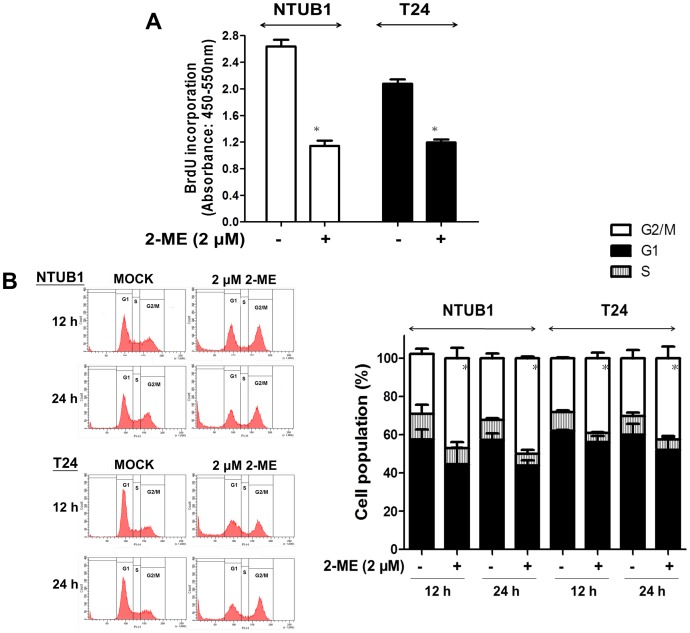
The effect of 2-ME on cell proliferation and cell cycle in UC cells. (A) NTUB1 and T24 cells were treated with 2-ME (2 µM) or DMSO (as non-treated control) for 24 h, cell proliferation were measured by BrdU incorporation assay. (B) NTUB1 and T24 cells were treated with mock (DMSO) or 2-ME (2 µM) for 12 h and 24 h, and cell cycle analyses were measured by flow cytometry. Quantitative data are presented as means ± SD of three independents experiments. * p<0.05 is interpreted to be significant as compared with control.

We then performed BrdU incorporation assay to further determine the effect of 2-ME on UC cell proliferation. As is shown in Figure 4A, 2-ME exerted significantly inhibitory effect on UC cell proliferation (p<0.05). Previous studies have shown that 2-ME could induce cell cycle arrest at the G2/M phase in various cancer cells [11], [13], [14], [15]. We furthermore determined the effects of 2-ME on cell cycle transition in human UC cells using flow cytometry. UC cells were treated with 2-ME (2 µM) or DMSO (as non-treated control) for 12 and 24 h. Exposure to 2-ME significantly increased the percentage of cell population at the G2/M phase at 12 and 24 h (p<0.05) (Figure 4B). The increase in the number of cells with sub-G_0_/G_1_ DNA contents at 12 and 24 h after 2-ME treatment also indicated the proapoptotic effects of 2-ME on UC cells.

To further clarify the underlying mechanism by which 2-ME induced G2/M cell cycle arrest in human UC cells, we analyzed the level of cell cycle regulatory proteins, including cdc25C, phospho-cdc25C (Thr48 and Ser216), cdc2, phospho-cdc2, cyclin A2, B1, D1, D3, E2, histone H3, phospho-histone H3 (Ser10), p27, CDK2, CDK4 by Western blot.

Since the maturation promoting factor (MPF) is made up of cdc2 and cyclin B, which promote entry into mitosis, activation of the cyclin B1/cdc2 function plays a critical role in G2/M transition. Cells with suppressed cyclin B1/cdc2 activity tend to be arrested in the G2 phase [25]. Cdc2 is inactivated by phosphorylation on Thr14 and Tyr15. Dephosphorylation of Thr14 and Tyr15 of cdc2, and hence activation of the cdc2·cyclin B1 kinase complex, is catalyzed by cdc25C [25], [26]. Cdc25C is a protein phosphatase responsible for dephosphorylating and activating cdc2, which is a crucial step in regulating the entry of all eukaryotic cells into mitosis [21]. The activity of cdc25C is regulated by phosphorylation [21]. When phosphorylated at Ser216, cdc25C binds to members of the 14-3-3 family of proteins and is sequestered in the cytoplasm, through which the function of cdc25C is negatively regulated. Conversely, phosphorylation at Thr48 activates the function of cdc25C [26].

**Figure 5:**
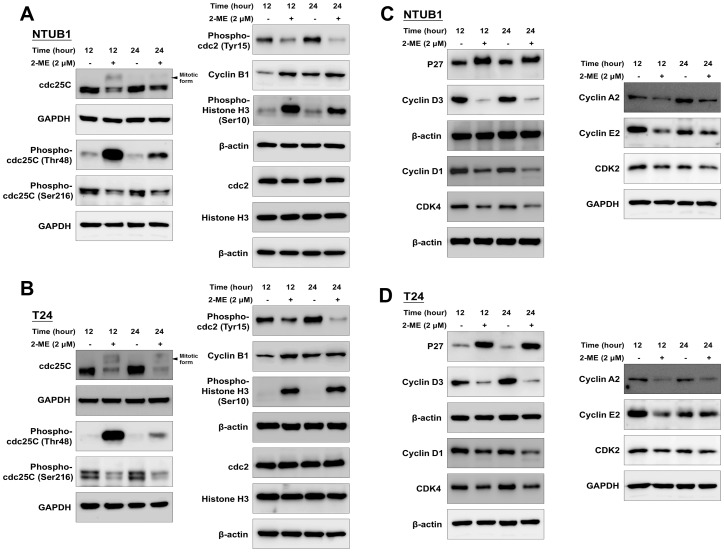
The effects of 2-ME on cell cycle regulatory proteins. NTUB1 and T24 cells were treated with 2ME (2 µM) for 12 and 24 h. The total cell lysates were analyzed for the levels of cell cycle regulatory proteins by Western blot using specific antibodies. (A) and (B) shows the levels of cdc25C, phospho-cdc25C (Thr48 and Ser216), phospho-cdc2 (Tyr15), cyclin B1, phospho-histone H3 (Ser10), cdc2, histone H3. Similarly, (C) and (D) shows the level of p27, cyclin D3, cyclin D1, CDK4, cyclin A2, cyclin E2, CDK2 proteins in NTUB1 and T24 cells after 2-ME treatment. Results shown are representative of at least three independent experiments.

Therefore, we examined the effects of 2-ME on the phosphorylations of cdc25 at Ser216 and Thr48. 2-ME increased the phosphorylation of cdc25C on Thr48 and decreased phosphorylation at Ser216 (Figure 5A and 5B). The presence of the cdc25C mitotic form was detected in NTUB1 and T24 cells at 12 h after the 2-ME treatment and decreased after 24 h (Figure 5A and 5B).

Based on these findings, it is reasonable to assume that 2-ME treatment led to decrease phosphorylated (inactive) cdc2 at Tyr15 and increased cyclin B1. We then confirmed this hypothesis by Western blot using an antibody specific for phospho-cdc2 (Tyr15) and cyclin B1 (Figure 5A and 5B). Additionally, site-specific phosphorylation of histone H3 on Ser10 appeared to occur exclusively during mitosis in mammalian cells [27]. Thus, we used phospho-histone H3 (Ser10) to determine the effect of 2-ME on progression into mitosis. We actually found upregulation of phospho-histone H3 (Ser10) after treatment with 2-ME. It is noteworthy that 2-ME exposure does not alter the levels of cdc2 and histone H3. All data mentioned above support that 2-ME induces cell cycle arrest at the mitotic phase.

As shown in Figure 5C and 5D, 2-ME reduces the protein levels of cyclin D1, CDK4, and cyclin D3 after 12 h and 24 h treatments. Moreover, 2-ME also induces the expression of p27 and the inhibition of cyclin D1 and CDK4. The decreased levels in cyclin A2, D3, D1, E2, CDK4, and CDK2 after the 2-ME treatment reflected cell cycle arrests at G2/M and failure to enter other phases. Moreover, 2-ME could not influence on cell proliferation and cell cycle regulators in SV-HUC cells (Figure S3). The quantitative analyses of relative cell cycle regulatory protein levels in UC cells treated with 2-ME versus DMSO (as non-treated control) are shown in Figure S2.

### The combination of As_2_O_3_ and 2-ME elicits a synergistic antitumor effect on the UC cells

**Figure 6:**
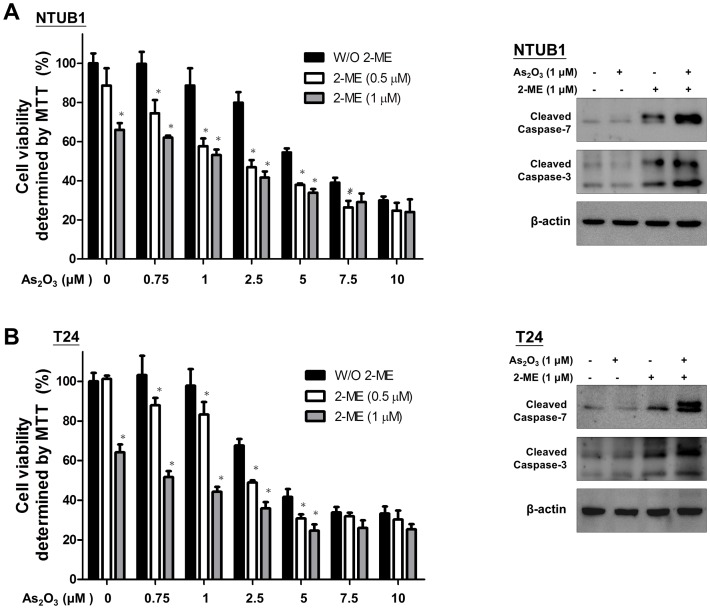
The combinative effects of 2-ME and As_2_O_3_ on cell viability and the level of cleaved caspase-3 and 7 in UC cells. (A) NTUB1 and (B) T24 cells were incubated in the presence of 2-ME (0.5 and 1 µM) and different concentration of As_2_O_3_ (0.75 to 10 µM) individually or in combination. Cell viability was measured by MTT assay. Quantitative analysis of cell viability are presented as means ± SD of three independents experiments. * p<0.05 is interpreted to be significant as compared with As_2_O_3_ treatment alone. The total cell lysates were harvested and analyzed by Western blot with specific antibodies against cleaved caspase-3 and 7 after treatment of 2-ME (1 µM), As_2_O_3_ (1 µM) and in combination. Results shown are representative of at least three independent experiments.

2-ME has been reported to enhance the antitumor effects of certain chemotherapeutic agents in various cancers [28]. Above all, the minimal toxicity in clinical use of 2-ME makes it promising for use in combination with current chemotherapeutic drugs. We then examined the combinative antitumor effects of 2-ME in UC cells with chemotherapeutic agents such as cisplatin, paclitaxel, and arsenic trioxide (As_2_O_3_). We found that no significantly synergistic antitumor effects existed after combination with cisplatin and paclitaxel (data not shown). However, the combination of As_2_O_3_ and 2-ME elicited synergistic cytotoxicity in the UC cells. The UC cells were incubated in the presence of 2-ME (0.5 and 1 µM), and different concentrations of As_2_O_3_ (0.75 to 10 µM) individually or in combination. As shown in Figure 6A and 6B, 2-ME and As_2_O_3_ cooperate to inducing cell death. The combination of 2-ME and As_2_O_3_ increased the inhibitory effect on the viability of UC cells as compared to single-agent treatments. The combination of As_2_O_3_ and 2-ME also markedly increased the cleavages of caspase-3 and -7 induced by single-agent treatments (Figure 6A and 6B). On the contrast, 2-ME, As_2_O_3_ and combination do not show effects on cell viability inhibition and apoptosis in SV-HUC cells (Figure S4A and 4B).

**Figure 7:**
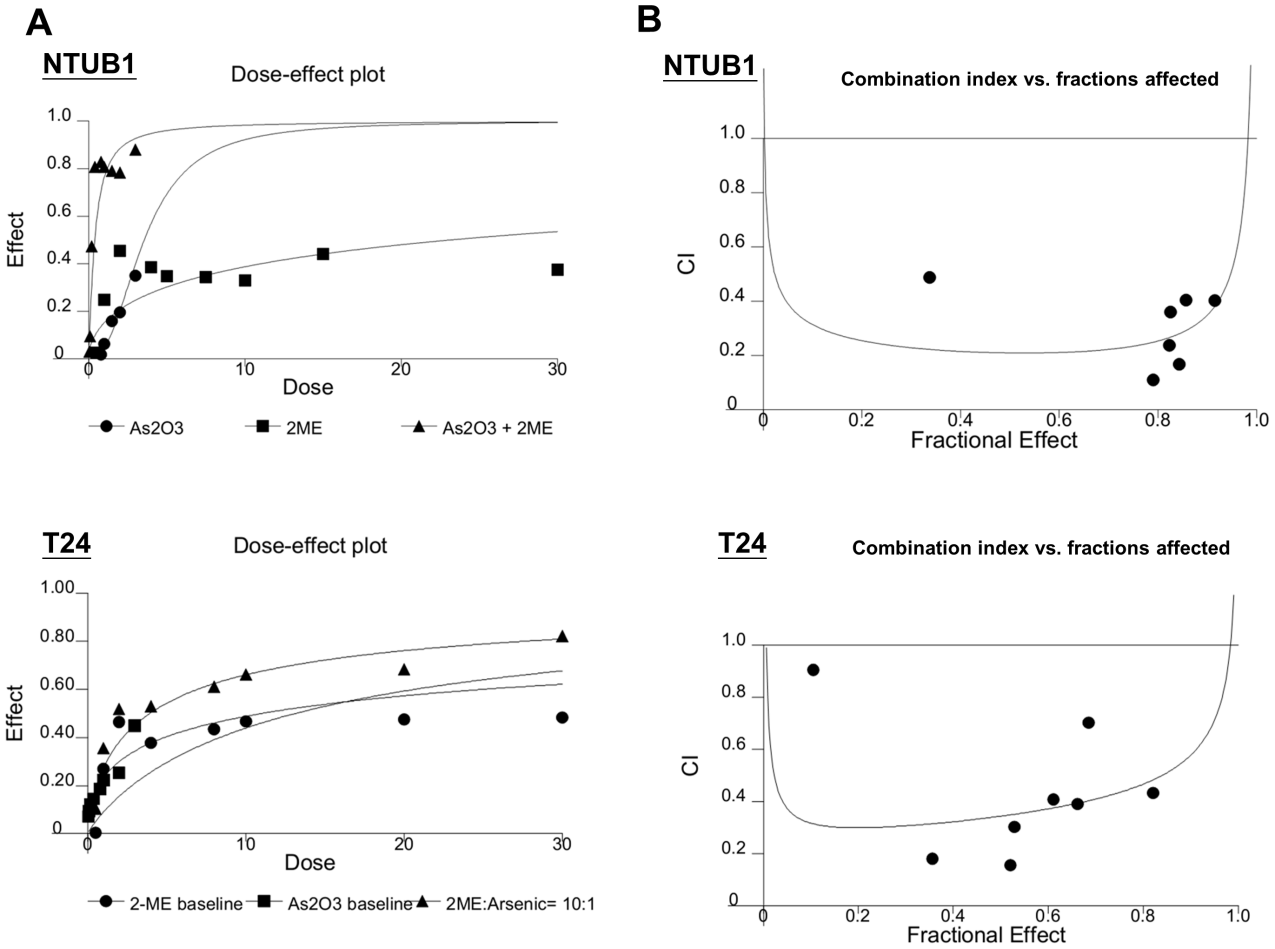
The combination with 2-ME and As_2_O_3_ at the ratio of 1∶ 10 induces synergistic inhibition on the viability of NTUB1 and T24 cells. UC cells were incubated in the presence of 2-ME, As_2_O_3_, and in combination at the concentration ratio of 1∶ 10. Cell viability was measured by MTT assay. Cell viability was measured by MTT after 24 h exposure. (A) The dose-effect plot reflects the dose-effect relationships for As_2_O_3_, 2-ME and the combination. (B) Combination index and fractions affected was plotted in combination with As_2_O_3_ and 2-ME.

We further evaluated the combinative drug effects using CalcuSyn software. The combinative effects of As_2_O_3_ and 2-ME at the concentration ratio of 10∶1 were subjected to the median-effect analysis using the mutually nonexclusive model. The effects of the combinations were then transformed into and displayed in a dose-effect plot (Figure 7A) and fraction affected-combination index plots (Figure 7B) in the two UC cell lines. The results showed that, in combination with As_2_O_3_, 2-ME could induce a synergistic antitumor effect (CI<1) at the concentration ratio of 10∶1.

## Discussion

2-ME is physiologically formed in small amounts in the human body [29]. It is known that the antiproliferative and proapoptotic activities in tumor cells are mediated independent of the estrogen receptors [5]. Previous studies have indicated that 2-ME elicited antitumor effects on various cancers [5], [8], [9], [10], [11]. Oral administration in human and animals has shown little or no toxicity at therapeutic doses [5], [7]. However, no study has explored the antitumor effect of 2-ME on human bladder UC cells until now. In this study, we confirmed that low-concentration 2-ME could effectively inhibit viability and induce apoptosis in bladder UC cells. Furthermore, 2-ME elicits minimal impact on the growth of transformed urothelial cells (SV-HUC). These findings reflect that 2-ME is a promising agent in treatment of advanced bladder UCs.

In terms of the underlying mechanisms of 2-ME–induced cytotoxicity, we first revealed that 2-ME–induced apoptosis is associated with DNA fragmentation and the phosphorylation of histone H2A.X. We also showed that the apoptotic effect of 2-ME is associated with down-regulation of phosphorylated Bad at Ser136 and 155.

Most of the previous studies reported that 2-ME induced cell cycle arrest at G2/M in other cancer cells. In this study, on the one hand, we consistently confirmed that 2-ME causes G2/M arrest in bladder UC cells by flow cytometry. On the other hand, we systemically analyzed the changes in the cell cycle regulatory proteins after the 2-ME treatment. The changes in the cell cycle regulatory proteins following 2-ME treatment in human breast cancer cells have been demonstrated [26], [30]. Choi et al. found that 2-ME can selectively induce mitotic prometaphase arrest in human breast cancer cells with a time-dependent upregulation of cyclin B1 and cdc2 proteins [26]. The 2-ME–induced prometaphase arrest is abrogated by selective knockdown of cyclin B1 and cdc2 or by pretreatment of the inhibitor of cyclin-dependent kinases. Similarly, our study further proved the changes in cell cycle regulatory proteins after 2-ME treatment are consistent with the findings of mitotic phase arrest.

Cdc25C is a protein phosphatase responsible for dephosphorylating and activating cdc2, which is a crucial step in regulating the entry of all eukaryotic cells into mitosis [25]. Phosphorylated at Ser216, cdc25C binds to members of the 14-3-3 family of proteins and is sequestered in the cytoplasm, through which the function of cdc25C is negatively regulated [31]; conversely, phosphorylation at Thr48 activates cdc25C [31]. Our study also shows consistent findings.

We also confirmed that phosphorylation of cdc2 at Tyr15 decreases in 2-ME–treated cells at 12 and 24 h. 2-ME treatments also upregulate cyclin B1 and phospho-histone H3 (Ser10), which will cause mitotic arrest. In agreement with previous findings, the decreased levels in cyclin D3, D1, A2, E2, CDK4, and CDK2, as well as the increased level of p27 after 2-ME treatment, also indicate cell cycle arrests at the mitotic phase and failure to enter other phases.

In a normal cell cycle, the transition from G2 phase to mitotic phase is triggered by the activation of the cyclin B1 and cdc2 complex. Cells with suppressed cyclin B1/cdc2 activity would tend to be arrested in the G2 phase; whereas cells with an elevated cyclin B1/cdc2 activity would be favored to enter and then proceed through mitosis. In the present study, 2-ME induced upregulation of cyclin B1 and cdc2 protein levels, but also altered its phosphorylation patterns. Collectively, these changes are expected to result in marked increases in the functionality of the cyclin B1/cdc2 complex. 2-ME causes disruption of microtubule formation in the mitotic phase [32]. Consequently, cells arrested in the mitotic phase would increase the level and activity of cyclin B1 and cdc2 to proceed through mitosis. As demonstrated by the study conducted by Choi et al., the early increase in cyclin B1 and cdc2 protein levels after 2-ME treatment are characteristic features of cells blocked in prometaphase [26].

2-ME has been reported to enhance the anticancer actions of microtubule-disrupting agents such as paclitaxel or docetaxel in breast cancer cells [33]. These microtubule inhibitors can bind to tubulins or microtubules and suppress microtubule dynamics and functions, subsequently inducing G2/M cell cycle arrest as well as apoptotic cell death. The potential application of 2-ME as an adjunct to enhance the anticancer actions of other commonly used chemotherapeutic agents is a promising strategy for treatment of bladder UC. In this study, we also tested the combinative effects of 2-ME with some clinically utilized chemotherapeutic agents such as cisplatin, As_2_O_3_, and paclitaxel. We observed that a synergistic antitumor effect exists only in combination with As_2_O_3._ Previous studies also indicated the cooperation between 2-ME and As_2_O_3_ mainly in leukemia cell model [34], [35], [36]. Our results reveal that 2-ME and As_2_O_3_ exerted low toxicity on SV-HUC cells. At the concentration, the combination can synergistically cooperate to inducing cytotoxicity against UC cells. .

## Conclusions

2-ME effectively induces the apoptosis of human UC cells and shows considerable promise as a therapeutic alternative in UCs. 2-ME significantly induces apoptosis through decreasing phospho-Bad and arresting the cell cycle at the mitotic phase in bladder UC cells. The synergistic apoptotic effect of combining 2-ME with As_2_O_3_ *in vitro* deserves further study for translation into the clinical environment.

## Supporting Information

Figure S1 **2-ME induces (A) phospho-histone H2A.X, (B) caspase activations and PARP cleavage in T24 cells instead of SV-HUC cells.** T24 and SV-HUC cells were treated by 2-ME (2 µM) for 24 h. The total cell lysates were harvested and analyzed by Western blot with specific antibodies against phospho-histone H2A.X, caspase-8, 9, cleaved caspase-3, 7 and PARP. CF is the abbreviation of cleaved form. Results shown are representative of at least three independent experiments.
(TIF)

Figure S2 **The quantitative analysis of relative cell cycle regulatory protein levels in UC cells.** (A) NTUB1 and (B) T24 cells treated with 2-ME versus DMSO (as non-treated control) for 12 and 24 h, the whole cell lysate were prepared and subjected to Western blot. The levels of target proteins were quantified by using Image J (NIH, USA) and normalized to each internal control. Protein levels are presented as mean±SD of three independent experiments. *p<0.05 is interpreted as significant in comparing 2-ME-treated to non-treated cells.
(TIF)

Figure S3 **2-ME exerted insignificant effects on cell proliferation and levels of cell cycle regulatory proteins in SV-HUC cells.** (A) SV-HUC cells were treated with 2-ME (2 µM) or DMSO (as non-treated control) for 24 h. Cell proliferation was measured by BrdU incorporation assay. (B) shows the levels of cell cycle regulatory proteins in SV-HUC cells after 2-ME treatment using Western blot. Results shown are representative of at least three independent experiments.
(TIF)

Figure S4 **2-ME does not appear to potentiate As_2_O_3_-induced cytotoxicity and activations of caspase-3 and 7 in SV-HUC cells.** (A) SV-HUC cells were incubated in the presence of 2-ME (0.5 and 1 µM) and various concentration of As_2_O_3_ (0.75 to 10 µM) individually or in combination for 24 h. Cell viability was measured by MTT assay. Quantitative analyses of cell viability are presented as means ± SD of three independents experiments. * p<0.05 is interpreted to be significant as compared with As_2_O_3_ treatment alone. (B) The total cell lysates were harvested and analyzed by Western blot with specific antibodies against cleaved caspase-3 and 7 after treatment of 2-ME (1 µM), As_2_O_3_ (1 µM) and in combination. Results shown are representative of at least three independent experiments.
(TIF)
