# Supplementary figures and images for: 2-Methoxyestradiol Induces Mitotic Arrest, Apoptosis, and Synergistic Cytotoxicity with Arsenic Trioxide in Human Urothelial Carcinoma Cells
Source: PLoS One. 2013 Aug 13;8(8):e68703. doi: 10.1371/journal.pone.0068703 (PMC3742604; doi:10.1371/journal.pone.0068703)

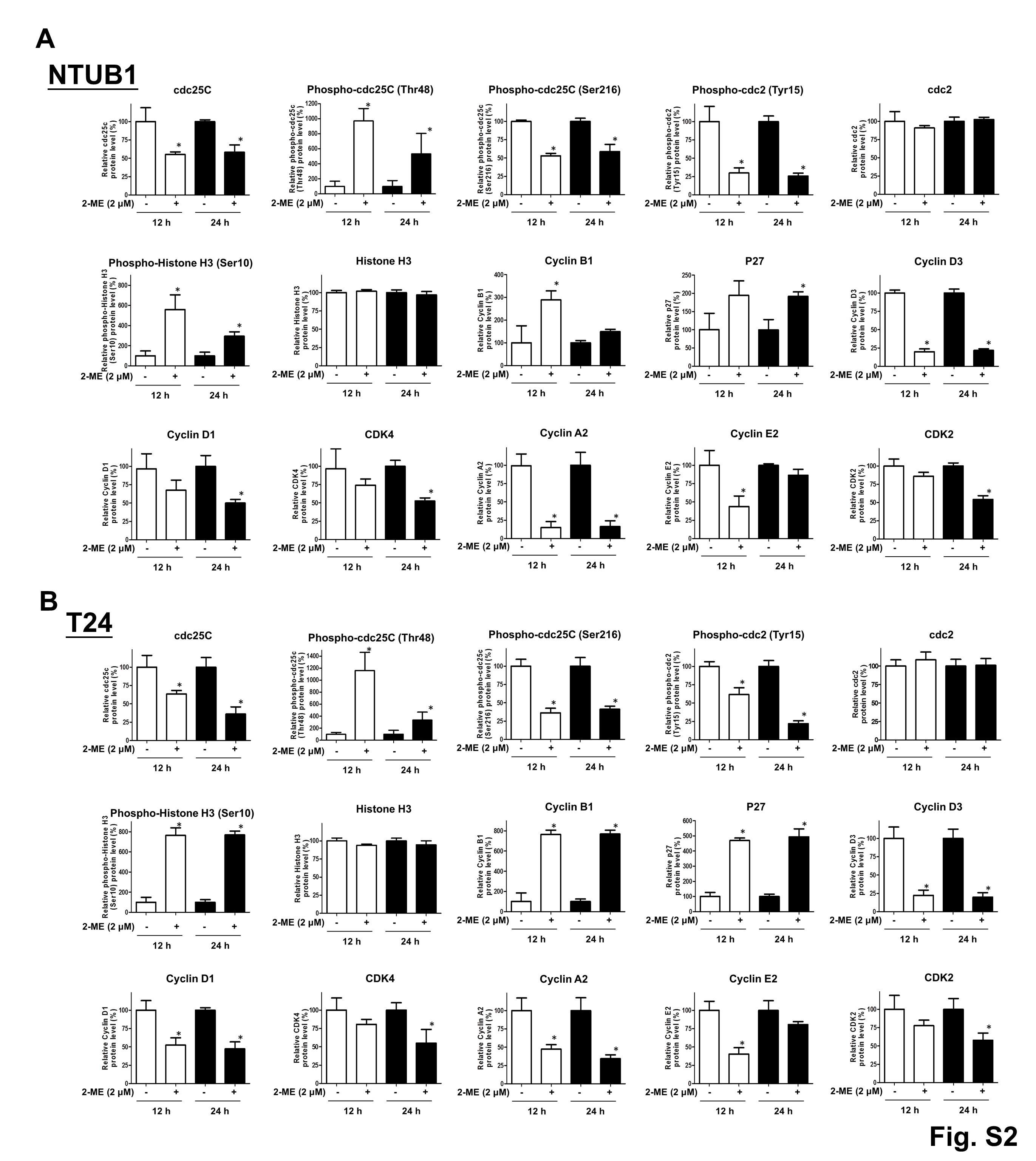

Supplement: Figure S2 — The quantitative analysis of relative cell cycle regulatory protein levels in UC cells. (A) NTUB1 and (B) T24 cells treated with 2-ME versus DMSO (as non-treated control) for 12 and 24 h, the whole cell lysate were prepared and subjected to Western blot. The levels of target proteins were quantified by using Image J (NIH, USA) and normalized to each internal control. Protein levels are presented as mean±SD of three independent experiments. *p<0.05 is interpreted as significant in comparing 2-ME-treated to non-treated cells. (TIF) [file pone.0068703.s002.tif]

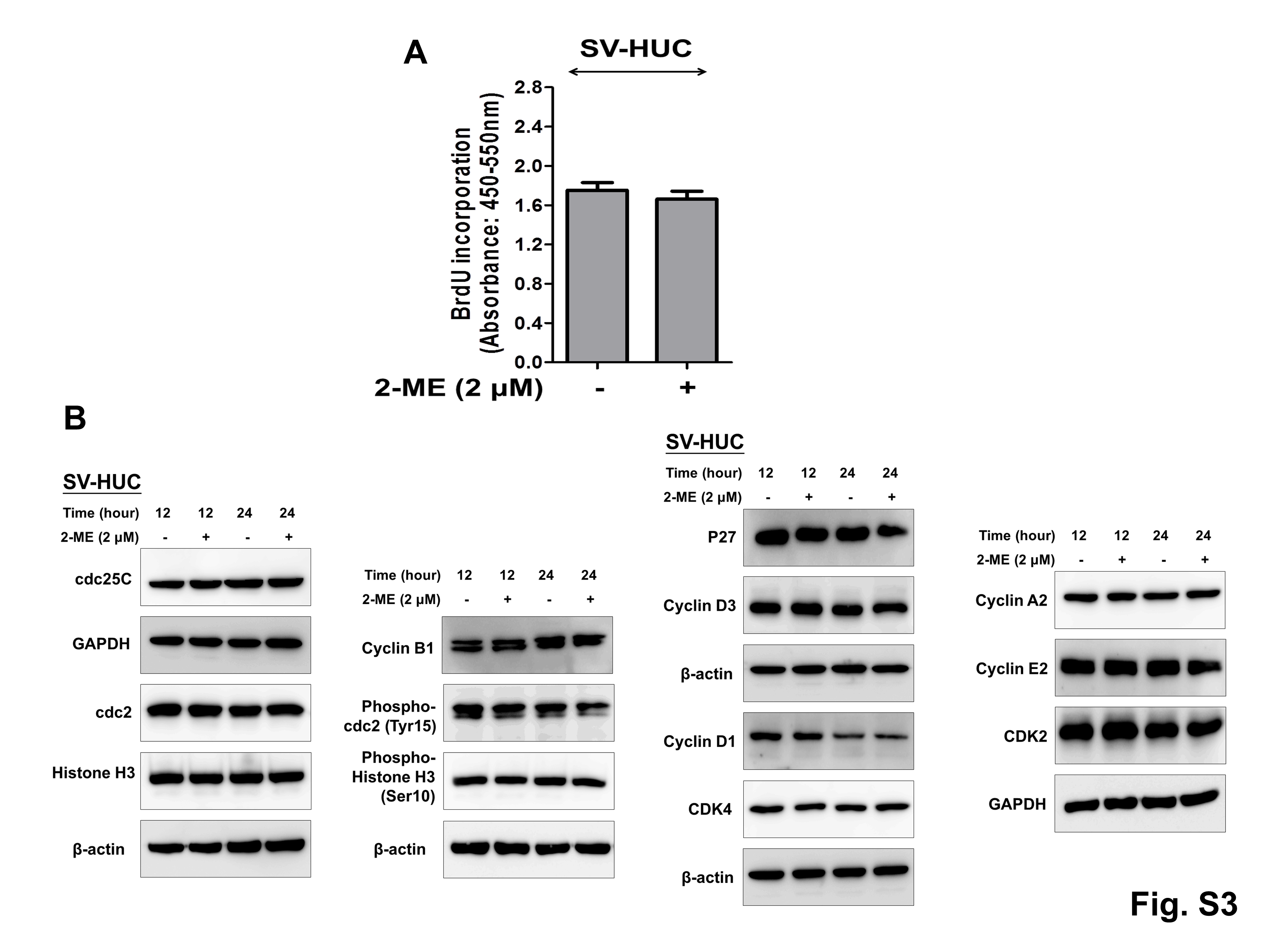

Supplement: Figure S3 — 2-ME exerted insignificant effects on cell proliferation and levels of cell cycle regulatory proteins in SV-HUC cells. (A) SV-HUC cells were treated with 2-ME (2 µM) or DMSO (as non-treated control) for 24 h. Cell proliferation was measured by BrdU incorporation assay. (B) shows the levels of cell cycle regulatory proteins in SV-HUC cells after 2-ME treatment using Western blot. Results shown are representative of at least three independent experiments. (TIF) [file pone.0068703.s003.tif]

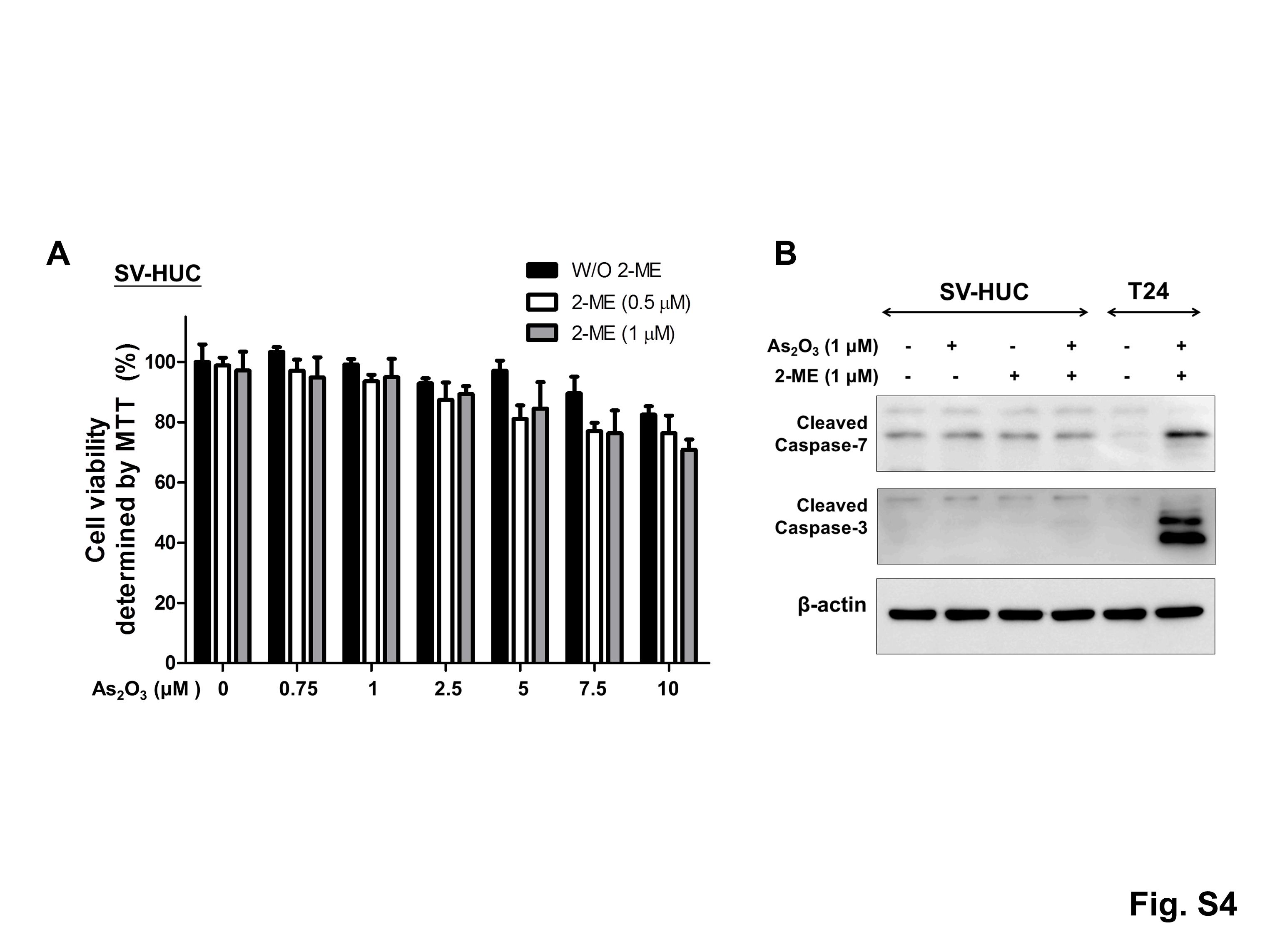

Supplement: Figure S4 — 2-ME does not appear to potentiate As2O3-induced cytotoxicity and activations of caspase-3 and 7 in SV-HUC cells. (A) SV-HUC cells were incubated in the presence of 2-ME (0.5 and 1 µM) and various concentration of As2O3 (0.75 to 10 µM) individually or in combination for 24 h. Cell viability was measured by MTT assay. Quantitative analyses of cell viability are presented as means ± SD of three independents experiments. * p<0.05 is interpreted to be significant as compared with As2O3 treatment alone. (B) The total cell lysates were harvested and analyzed by Western blot with specific antibodies against cleaved caspase-3 and 7 after treatment of 2-ME (1 µM), As2O3 (1 µM) and in combination. Results shown are representative of at least three independent experiments. (TIF) [file pone.0068703.s004.tif]
